# 超高效液相色谱-串联质谱法鉴定驼乳及其制品中的动物源性成分

**DOI:** 10.3724/SP.J.1123.2023.07027

**Published:** 2024-01-08

**Authors:** Shuqing GU, Niannian CHEN, Jing ZENG, Xiaoyu PENG, Min ZHANG, Yu GAO, Lina PAN, Cheng GE, Wei LI, Xionghai YI, Dehua GUO, Xiaojun DENG

**Affiliations:** 1.上海海关动植物与食品检验检疫技术中心, 上海 200135; 1. Technical Center for Animal Plant and Food Inspection and Quarantine, Shanghai Customs, Shanghai 200135, China; 2.澳优乳业(中国)有限公司, 湖南 长沙 410127; 2. Ausnutria Dairy (China) Co., Ltd., Changsha 410127, China; 3.上海体育大学国家兴奋剂检测上海实验室, 上海 200438; 3. National Doping Testing Shanghai Laboratory, Shanghai University of Sports, Shanghai 200438, China

**Keywords:** 液相色谱-串联质谱, 骆驼乳, 骆驼奶粉, 掺假定量, liquid chromatography-tandem mass spectrometry (LC-MS/MS), camel milk, camel milk powder, adulteration quantification

## Abstract

采用蛋白质组学技术建立了驼乳及其制品中的动物乳源性成分特异性肽生物标志物的鉴定方法。样品经脱脂、蛋白质提取、胰蛋白酶水解后,利用超高效液相色谱-四极杆/静电场轨道阱高分辨质谱仪(UHPLC-Q/Exactive-HRMS)和Protein Pilot软件,实现了多肽生物标记物的鉴定;然后通过基本局部比对搜索工具(BLAST)与Uniprot数据库对比分析,筛选出了骆驼、家牛、水牛、牦牛、山羊、绵羊、驴和马共8个物种的22条肽生物标志物;最后利用超高效液相色谱-三重四极杆质谱(UHPLC-QqQ-MS)系统对这22条特征性多肽进行验证,采用多反应监测(MRM)模式建立了定量方法。实验优化了骆驼奶中酪蛋白的预处理方法,如冷冻脱脂、沉淀蛋白试剂和复溶液的选择等,并建立了基于生物标志物肽测定牛和山羊奶/奶粉掺假骆驼奶/奶粉的无标记定量方法。将牛、山羊和骆驼奶/奶粉等比例混合,分别对牛和山羊的特征肽段进行检测,结果显示,该方法对掺假液体乳/固体奶粉样品显示出良好的线性关系,抗干扰能力强,灵敏度高,重复性好,液态奶和奶粉的掺杂结果均与理论值接近。另外,该方法还被应用于11种骆驼奶和奶粉中家牛、水牛、牦牛、山羊、绵羊、驴和马乳源性成分的鉴别,为骆驼乳及乳制品中多种动物乳源性成分的鉴别提供了有效的技术平台。

## Abstract (en)

A method for identifying specific peptide biomarkers of animal-milk-derived components in camel milk and its products was established using proteomics. Samples were prepared by defatting, protein extraction, and trypsin hydrolysis, and proteins and peptides were identified using ultra-high performance liquid chromatography-quadrupole/electrostatic orbitrap-high resolution mass spectrometry (UHPLC-Q/Exactive-HRMS) and Protein Pilot software. Twenty two peptide biomarkers from eight species (i.e., *Camelus*, *Bos taurus*, *Bubalus bubalis*, *Bos grunniens/Bos mutus*, *Capra hircus*, *Ovis aries*, *Equus asinus*, *Equus caballus*) were identified by comparing the basic local alignment search tool (BLAST) with the Uniprot database. Verification of these marker peptides were performed quantitatively using a UHPLC-triple-quadrupole mass-spectrometry (QqQ-MS) system by multiple reaction monitoring (MRM). The pretreatment method of casein in camel milk was optimized, such as defatting, protein precipitation, and re-dissolving buffer solution. The effects of various mass-spectrometry parameters, such as atomization gas, heating- and drying-gas flow rates, and desolvation-tube (DL) and ion-source-interface temperatures on ion-response intensity were optimized. Camel milk signature peptides were detected in a mixture of milk from other seven species to ensure specificity for the selected biomarker peptides. The signature peptides of seven other species were also detected in camel milk. No mutual interference between the selected biomarker peptides of the various species was observed. Adulterated camel milk and milk powder were also quantitatively studied by adding 0, 2.5%, 5%, 10%, 25%, 50%, 75%, and 100% bovine milk or goat milk to camel milk. Similarly, the same mass proportion of bovine milk powder or goat milk powder was added to camel milk powder. A quantitative standard curve for adulteration was constructed by plotting the peak areas of characteristic cow or goat peptide segments in each mixed sample against the mass percentage of the added adulterant. The adulteration standard curves exhibited good linearity, with correlation coefficients (*r*^2^) greater than 0.99. The limits of detection and quantification (LODs and LOQs, respectively) of the method were determined as three- and ten-times the signal-to-noise ratio (*S/N*). The minimum adulteration LODs of bovine milk and goat milk in camel milk were determined to be 0.35% and 0.49%, respectively, and the minimum LOQs were 1.20% and 1.69%, respectively. The minimum adulteration LODs of bovine milk powder and goat milk powder in camel milk powder were determined to be 0.68% and 0.73%, respectively, and the minimum LOQs were 1.65% and 2.45%, respectively. The accuracy of the adulteration quantification method was investigated by validating the quantitative detection results for 1∶1∶1 (mass ratio) mixtures of camel milk, bovine milk, and goat milk, as well as camel-milk powder, bovine milk powder, and goat-milk powder, which revealed that this method exhibits good linearity, strong anti-interference, high sensitivity, and good repeatability for adulterated liquid-milk/solid-milk-powder samples. The adulteration results for both liquid milk and milk powder are close to the theoretical values. Finally, 11 actual commercially available samples, including five camel-milk and six camel-milk-powder samples were analyzed, which revealed that only camel signature peptides were detected in 10 samples, while camel and bovine signature peptides were both detected in one camel-milk-powder sample. The ingredient list of the latter sample revealed that it contained whole milk powder from an unidentified source; therefore, we infer that the bovine signature peptides originate from the whole milk powder. These signature peptides also demonstrate the necessity and practical significance of establishing this identification method.

目前,动物乳市场朝着多元化方向快速发展,除普通牛乳外,骆驼乳、山羊乳、水牛乳、马乳、驴乳、牦牛乳等特种乳受到人们的广泛关注。联合国粮农组织(Food and Agriculture Organization, FAO)的数据显示,截止到2020年,牛乳仍占全球生鲜乳生产量排名的榜首,水牛奶、山羊奶、绵羊奶和骆驼奶等特种乳产量相对较低,其中骆驼乳产量远低于其他动物乳^[1]^。驼乳营养丰富,被誉为“沙漠白金”,不含有过敏原*β*-乳球蛋白,适合过敏症患者,且成分与人乳相似,可以作为人乳替代品^[2]^。由于骆驼乳产量低,乳供应量明显受季节性波动的影响,且奶源质量难以控制,骆驼乳及其制品成为蓄意掺假的目标。向高价值的骆驼乳及其制品中掺入低价值的牛、羊等动物乳是最常见的掺假行为,这不仅会造成消费者的经济损失,还可能危害消费者的身体健康。因此,亟需建立一种准确、高效的驼乳及其制品中的动物源性成分测定方法。

常用的骆驼奶和其他物种奶的鉴别主要采用基于DNA的聚合酶链式反应(PCR)^[3⇓⇓⇓-7]^、基于蛋白质组学的质谱(MS)^[8⇓-10]^、二维凝胶电泳(2-DE)^[11]^与核磁共振(NMR)^[12]^等方法。然而,由于多重引物设计要求高,PCR方法无法实现8个物种同时检测;2-DE方法分辨率较低,需要结合MS方法进行验证,这两种方法均增加了实验的复杂性;而NMR方法无法靶向识别目标物,需要额外的数据解析。基于蛋白质组学的质谱多反应监测(MRM)技术克服了以上问题,具有通量高、特异性强且操作简便的优势,可根据不同物种本身蛋白质氨基酸序列的细微差异识别动物乳来源,同时可以高效、准确地靶向定量多个物种的特征肽段^[13]^。

本文将超高效液相色谱-四极杆/静电场轨道阱高分辨质谱(UHPLC-Q/Exactive-HRMS)和超高效液相色谱-三重四极杆质谱(UHPLC-QqQ-MS)相结合,筛选出骆驼、家牛、水牛、牦牛、山羊、绵羊、驴和马共8个物种的22个特征肽段,并建立了以特征肽段为分析对象的骆驼奶/奶粉掺假鉴别和定量方法。

## 1 实验部分

### 1.1 仪器、试剂与材料

Ultimate 3000超高效液相色谱-Q-Exactive HRMS四极杆/静电场轨道阱高分辨质谱仪(美国Thermo Fisher公司);LC-30 AD超高效液相色谱-MS-8060三重四极杆质谱仪(日本Shimadzu公司);离心机(Allegra X-22R,美国Beckman Coulter公司);水浴锅(SW22,德国Julabo公司);涡旋混合器(美国Talboys公司);所有实验用水由Milli-Q超纯水系统(美国Millipore公司)制得。

碳酸氢铵(NH_4_HCO_3_,纯度≥99%)、尿素(CH_4_N_2_O,纯度≥99%)、氯化钠(NaCl,纯度≥99%)、氯化钙(CaCl_2_,纯度≥99%)、二硫苏糖醇(DTT,纯度≥99%)、碘乙酰胺(IAA,纯度≥99%)均购自德国Merck公司。乙腈(色谱纯,美国Thermo Fisher公司)、甲酸(色谱纯,德国Fluka公司)、Tris-HCl缓冲液(1 mol/L, pH=8.0,上海碧云天生物技术有限公司)、胰蛋白酶(测序级,美国Promega公司)。

低蛋白吸附管(1.5 mL,德国Eppendorf公司),低蛋白吸附移液器吸头(10~100 μL, 100~1000 μL,德国Brand公司),醋酸纤维素膜(0.22 μm,天津博纳艾杰尔科技有限公司)。骆驼奶/奶粉及其他动物奶/奶粉购自市场。

### 1.2 实验条件

#### 1.2.1 样品前处理

吸取10 mL液态乳(或称取1 g奶粉用超纯水定容至10 mL),于-20 ℃冷冻10 min, 10000 r/min下离心5 min,然后用玻璃棒挑去液态乳上层的脂肪层。取200 μL脱脂乳,加入800 μL乙腈(含1%甲酸),振荡混匀,-20 ℃放置30 min。10000 r/min下离心5 min,弃去上清液。加入1 mL超纯水悬浮沉淀并振荡混匀,10000 r/min下离心3 min,弃去上清液。向沉淀中加入800 μL 50 mmol/L NH_4_HCO_3_溶液(含6 mol/L尿素),涡旋振荡5 min。观察无明显颗粒,用50 mmol/L NH_4_HCO_3_溶液(含6 mol/L尿素)定容至1 mL。用超纯水稀释5倍后待酶解。

#### 1.2.2 酶解

吸取200 μL试样于1.5 mL低蛋白吸附离心管中,加入200 μL 500 mmol/L NH_4_HCO_3_溶液,10 μL 500 mmol/L DTT,涡旋混匀,于75 ℃恒温水浴中振荡30 min。静置至室温,加入20 μL 500 mmol/L IAA,混匀,置于暗处避光反应30 min。加入10 μL 100 mmol/L CaCl_2_溶液,向离心管中加入20 μL 100 μg/mL的胰蛋白酶溶液,37 ℃酶解过夜。放至室温,加入10 μL甲酸终止反应,涡旋后静置15 min。加水补至1 mL, 用0.22 μm低蛋白吸附滤膜过滤,滤液待上机检测。

#### 1.2.3 UHPLC-Q/Exactive-HRMS条件

采用UHPLC-Q/Exactive-HRMS进行多肽生物标志物的鉴定。

色谱条件 色谱柱为Aeris peptide XB-C18柱(100 mm×2.1 mm, 1.7 μm, Phenomenex, USA),流动相A为0.1%(v/v)甲酸水溶液,B为0.1%(v/v)甲酸乙腈溶液。液相色谱梯度洗脱程序:0~4 min, 97%A; 4~19 min, 97%A~30%A; 19~20 min, 30%A~10%A; 20~24 min, 10%A; 24~25 min, 10%A~97%A; 25~30 min, 97%A。流速:0.2 mL/min;进样体积:10 μL;柱温:40 ℃。

质谱条件 电喷雾电离(ESI)源,正离子模式;喷雾电压3.5 kV,毛细管温度320 ℃,辅助气体温度250 ℃,鞘气压力206.85 kPa,辅助气压力68.95 kPa;采用全扫描数据依赖的二级离子扫描模式(Full MS/Data-dependent-MS^2^),扫描范围为*m/z* 300~1500 ;全扫描一级质谱和二级质谱的分辨率分别为70000和17500。

#### 1.2.4 UHPLC-QqQ-MS条件

采用UHPLC-QqQ-MS定量检测特征肽段。

色谱条件 XBridge BEH Amide XP色谱柱(100 mm×2.1 mm, 1.7 μm),流动相A为0.1%(v/v)甲酸乙腈溶液,流动相B为0.1%(v/v)甲酸水溶液。梯度洗脱程序:0~2 min, 5%A; 2~11 min, 5%A~55%A; 11~13 min, 55%A; 13~13.1 min, 55%A~5%A; 13.1~15 min, 5%A。流速:0.3 mL/min,进样体积:5 μL;柱温:40 ℃。

质谱条件 ESI源,正离子模式;雾化气:氮气,2 L/min;加热气:空气,12 L/min;干燥气:氮气,6 L/min;碰撞气:氩气;离子源接口温度:350 ℃;脱溶剂管(DL)温度:300 ℃;加热模块温度:400 ℃。

## 2 结果与讨论

### 2.1 特征肽段的选择

特征肽段的筛选原则与步骤和之前的工作^[14]^所述一致。采用Uniprot数据库对骆驼(*Camelus*)、家牛(*Bos taurus*)、水牛(*Bubalus bubalis*)、牦牛(*Bos grunniens/Bos mutus*)、山羊(*Capra hircus*)、绵羊(*Ovis aries*)、驴(*Equus asinus*)和马(*Equus caballus*)这8个物种搜索常见乳蛋白(酪蛋白、*α*-乳球蛋白和*β*-乳清蛋白等)的氨基酸序列。借助Skyline软件模拟酶切后,基于BLAST搜索UniprotKB/Swiss-Prot数据库,筛选理论特征肽段。采用UHPLC-Q/Exactive-HRMS对8个物种来源的真实奶/奶粉进行蛋白质确证。选Full MS/Data-dependent-MS^2^模式进行扫描,通过Protein Pilot软件(Version5.0, ABSCIEX)对特征肽段进行鉴定和筛选。多肽的质谱鉴定主要考察特征肽段的质谱碎片离子检测结果与理论碎片的匹配度。检测到的碎片离子峰和理论碎片峰匹配度高,表明该肽段的置信度高,更适合于后续的MRM定量分析。多肽在二级质谱中,主要产生C端碎片离子(y离子)和N端碎片离子(b离子)。以骆驼特征肽段INEDNHPQLGEPVK碎片离子质谱图(图1)为例,理论y离子碎片峰和b离子碎片峰与实验获得的碎片峰具有较好的匹配度,28个理论y、b离子碎片峰中有19个碎片峰得到鉴定,表明该肽段的实验结果与理论碎裂模式高度匹配,可进一步应用于MRM模式定量分析。

**图1:**
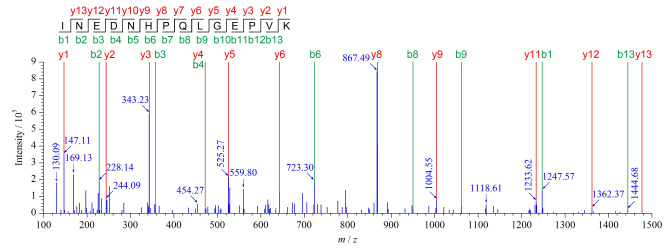
骆驼特征肽段INEDNHPQLGEPVK(*m/z* 795.4)的碎片离子实验结果与理论对比

选择鉴定结果较好的肽段作为备选特征肽段。特征肽段为目标蛋白中所特有的能够将其与其他蛋白质特异性区分的肽段序列,是一段高保守的氨基酸序列,不含易脱氨的Asp-Gly、易环化的N端Gln等,不易发生修饰影响定量;易被质谱系统检测,且具有响应较强的碎片离子;酶切具有较高的重现性,不会发生错切或漏切;8~25个氨基酸长度的肽为优先选择。在满足上述条件的基础上,还要考察特征肽段在实际样品检测中的适用性,采用MRM技术对多种真实物种来源的样品乳酶切后进行质谱检测采集数据,保留响应高、干扰峰少、稳定的肽段,最终确定骆驼、家牛、水牛、牦牛、山羊、绵羊、驴和马共22条特征肽段,结果见表1。

**表1:** 8个物种的特征肽段及MRM参数

Animal	Uniprot ID	Protein		Peptide No.	Peptide		t_R_/ min	Precursor ion (m/z)	Product ion (m/z)	CE/ eV
Camelus	O97943	α-S1-casein		C1	^151^INEDNHPQLGEPVK^164^		5.16	795.4 (2+)	1004.6^*^	31.3
									1061.5	30.3
									1118.5	27.3
	P79139	κ-casein		C2	^54^YPSYGINYYQHR^65^		5.77	780.9 (2+)	1050.5	29.7
									880.4	32.7
									766.4	32.7
				C3	^66^LAVPINNQFIPYPNYAKPVAIR^87^		7.26	833.5 (3+)	1128.7^*^	35.0
									917.6	38.0
									754.5	38.0
	Q9TVD0	β-casein		C4	^50^IEEQQQTEDEQQDK^63^		4.09	874.4 (2+)	992.4^*^	35.5
									891.4	34.5
									762.3	32.5
Bos taurus	P02662	α-S1-casein		B1	^120^VPQLEIVPNSAEER^133^		6.41	790.9 (2+)	802.4	32.1
									901.4	32.1
									567.3	30.1
	P02663	α-S2-casein		B2	^188^FALPQYLK^195^		6.84	490.3 (2+)	648.4	17.1
									761.5	16.1
									832.5	17.1
	P02668	κ-casein		B3	^89^SPAQILQWQVLSNTVPAK^106^		7.55	990.5 (2+)	315.2^*^	40.1
									384.2	43.1
									497.3	40.1
Bubalus bubalis	unsigned	unsigned		Bub1	AFKPTELGEVITK		6.49	716.9 (2+)	460.3	30.2
									589.4	30.2
									888.5	30.2
	Q9TSI0	β-casein		Bub2	^63^IHPFAQTQSLVYPFPGPIPK^82^		7.39	746.4 (3+)	852.5^*^	22.8
									1015.6	22.8
									1114.6	23.8
Bos grunniens/	A0A344X7B8	α-S2-casein		M1	^203^AMEPWIQPK^211^		5.61	550.3 (2+)	897.5	21.5
Bos mutus									768.4^*^	21.5
									485.3	25.5
	A0A344X7C0	κ-casein		M2	^89^SPAQILQWQVLSSTVPAK^106^		6.62	651.7 (3+)	802.5	31.3
									689.4	33.3
									610.4	31.3
Capra hircus	P33049	α-S2-casein		G1	^107^FPQYLQYPYQGPIVLNPWDQVK^128^		8.18	898.5 (3+)	999.5	19.6
									886.4	20.3
									772.4^*^	21.3
	P02670	κ-casein		G2	^89^SPAQTLQWQVLPNTVPAK^106^		7.02	989.5 (2+)	938.6	36.1
									839.5	36.1
									726.4	35.1
Ovis aries	P67976	β-lactoglobulin	S2			^142^TPEVDNEALEK^152^	5.21	622.8 (2+)	1046.5	25.4	
									917.5	25.4	
									818.4^*^	24.4	
Equus asinus	P86272	α-S1-casein	D1			^110^YNQLQLQAIYAQEQLIR^126^	7.47	698.0 (3+)	1020.5^*^	24.0	
									857.5	22.0	
									786.4	22.0	
	CAV00691.1	α-S2-casein	D2			^139^TGASPFIPIVNTEQLFTSEEIPK^161^	8.30	840.1 (3+)	950.5	26.2	
									803.4	21.2	
									702.4	24.2	
	P19647	β-lactoglobulin-2	D3			^139^ALQPLPGHVQIIQDPSGGQER^159^	6.29	747.4 (3+)	973.4	22.8	
									845.4	23.8	
									730.3	30.8	
Equus caballus	D2KAS0	α-S2-casein	H1			^122^TGDSPFIPIVNTEQLFTSEEIPK^144^	8.31	854.8 (3+)	950.5	26.8	
									803.4	27.8	
									718.3	23.8	
	Q9GKK3	β-casein	H2			^204^DTPVQAFLLYQDPR^217^	7.62	831.9 (2+)	904.5	28.8	
									791.4	26.8	
									678.3	26.8	
			H3			^218^LGPTGELDPATQPIVAVHNPVIV^240^	7.56	779.8 (3+)	1157.7^*^	25.0	
									947.6	25.0	
									848.5	24.0	
									777.5	23.0	
			H4			^124^LQEITVIPK^132^	6.25	520.8 (2+)	799.5	17.3	
									670.4	18.3	
									557.4	18.3	
	P07380	β-lactoglobulin-2	H5			^165^VQIVQDPSGGQER1^77^	5.20	706.9 (2+)	1072.5	25.8	
									973.4	24.8	
									730.3	23.8	

*Quantitative ion; CE: collision energy.

### 2.2 MRM方法的优化

在质谱电离技术中,所谓的“软电离”技术,例如电喷雾电离,灵敏度高,源内碎片最少,已发展成为食品蛋白质和多肽分析的首选方法^[15,16]^。通过优化仪器的碰撞能量、载气流量、脱溶剂管温度和离子源接口温度,可以提高方法的灵敏度。以骆驼特征肽段IEEQQQTEDEQQDK(874.4^2+^→ 992.4^+^)为例,子离子是由母离子经氩气碰撞后所产生的碎片,每个子离子均对应一个最佳的碰撞能,利用Skyline软件生成优化碰撞能方法,对离子通路*m/z* 874.4>992.4分别施加29.5~39.5 eV的11个不同碰撞能,结果表明,碰撞能为35.5 eV时可获得最佳的响应强度(图2a)。

**图2:**
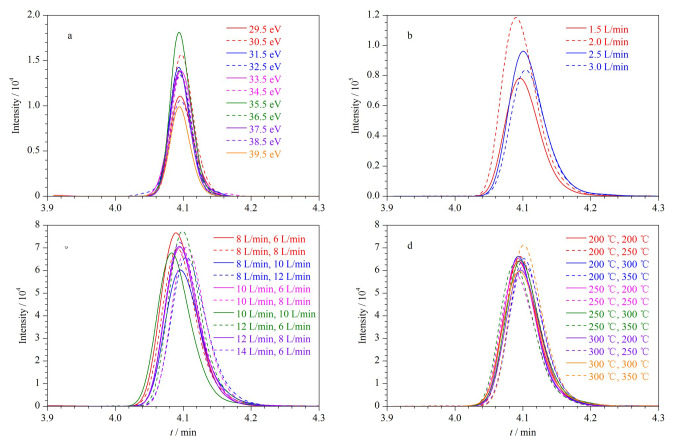
骆驼特征肽段IEEQQQTEDEQQDK的质谱条件考察

利用岛津LC-MS/MS自带的Interface软件考察了雾化气、加热气和干燥气流量、脱溶剂管温度和离子源接口温度对离子响应强度的影响,如图2b~d所示。结果表明,当雾化气、加热气和干燥气流量分别为2、12和6 L/min时,离子响应强度最高;当DL温度和离子接口温度分别为300 ℃和350 ℃时,离子响应强度最佳。按照相同步骤,对每个离子对优化出一个最佳的质谱条件,生成最终的MRM定量方法。22条特征肽段优化后的MRM参数见表1,优化后特征肽段的提取离子色谱图见图3。

**图3:**
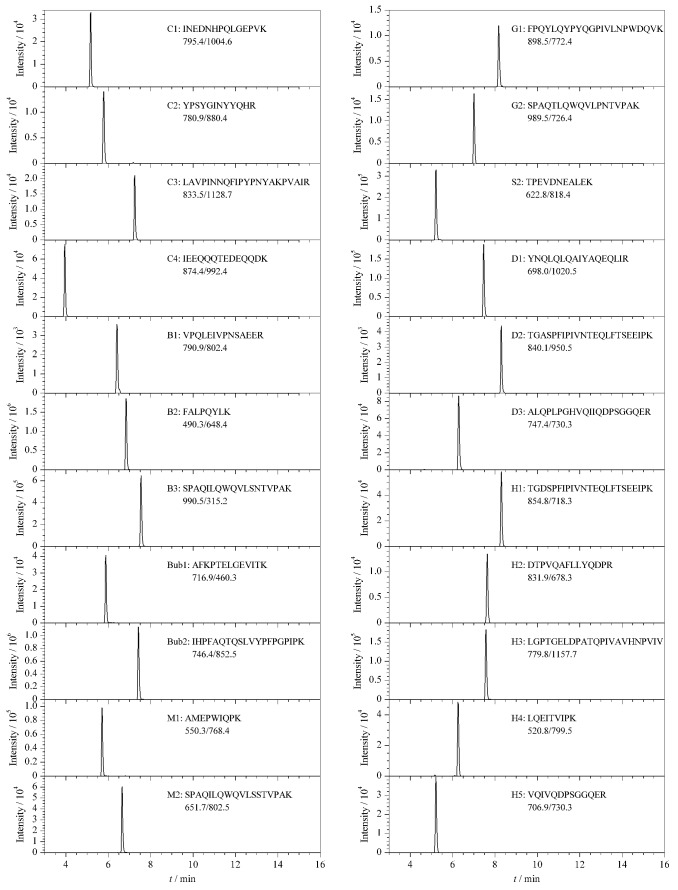
22个特征肽段的提取离子流色谱图

### 2.3 前处理条件的选择

全脂奶主要由乳脂球和酪蛋白胶束组成,酪蛋白胶束粘附在乳脂球表面,脱脂处理后二者分离^[17]^。有研究表明,部分酪蛋白通过疏水键与胶束结合,冷却至4 ℃会使一些键断裂,导致*β*-酪蛋白从胶束中解离^[18]^,增加其游离态浓度。因此,本工作对比了酪蛋白提取前脱脂处理前后质谱响应信号的变化情况,结果显示冷冻脱脂确实对*β*-酪蛋白肽段C4的响应信号显示出积极作用(图4a)。

**图4:**
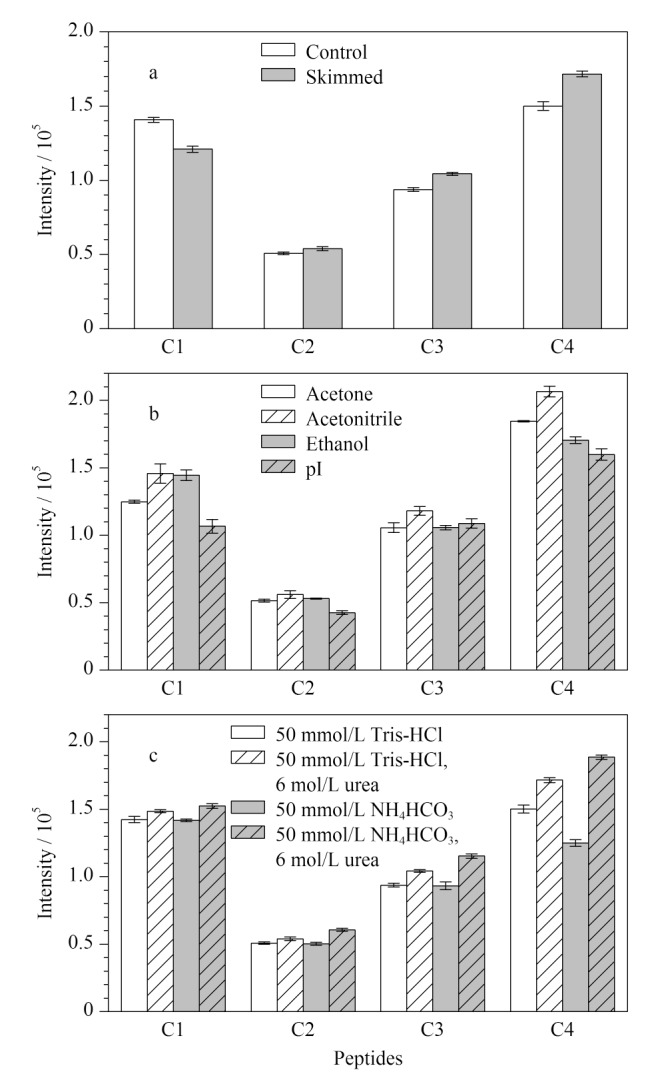
不同前处理方法对4种肽段响应强度的影响(*n*=3)

动物乳中酪蛋白含量约占蛋白质总量的80%左右^[19]^,对酪蛋白进行提取纯化有利于去除杂质,增加所选定量肽段的检测信号。本工作综合考察了丙酮、乙腈、乙醇3种不同的有机溶剂沉淀法和等电点(isoelectric point, pI)沉淀法对肽段响应强度的影响、操作过程的难易程度、沉淀后蛋白质的复溶性。结果表明,乙腈沉淀法操作简便、易复溶,经该方法提取的酪蛋白特征肽段响应强度最高(图4b)。孙姗姗等的研究^[20]^表明等电点沉淀法优于有机溶剂沉淀法,乙腈沉淀法会使蛋白质变性而降低复溶率。但等电点沉淀法需过夜处理,通量较低。本研究将乙腈沉淀法在低温下进行,可在一定程度上保护蛋白质不变性;提取沉淀过程仅需30 min,较等电点沉淀法表现出更好的提取效果,因此采用乙腈沉淀法提取驼乳中的酪蛋白。

酪蛋白有很强的形成大分子聚集体(即酪蛋白胶束)的倾向,超速离心得到的酪蛋白沉淀难以复溶^[21]^。复溶效率不仅影响定量结果,也关系到前处理操作的难易程度与实验时间。高浓度尿素溶液和高pH值条件有利于酪蛋白胶束解离^[22]^。本研究比较了pH=8.0±0.1的50 mmol/L Tris-HCl缓冲液、含6 mol/L尿素的50 mmol/L Tris-HCl缓冲液、50 mmol/L NH_4_HCO_3_缓冲液、含6 mol/L尿素的50 mmol/L NH_4_HCO_3_缓冲液对酪蛋白沉淀后复溶回收率的影响,结果如图4c所示。在相同的pH下,与缓冲液中不加入尿素相比,缓冲液中加入尿素能够提高复溶率,可能是由于尿素作为蛋白质变性剂提高了蛋白的溶解性。最终选择含6 mol/L尿素的50 mmol/L NH_4_HCO_3_缓冲液作为复溶液。

### 2.4 特异性验证

虽然所筛选的各物种特征肽段均经过BLAST搜索确定在数据库中具有特异性,但是某些物种的数据库存在不完善的情况^[23]^;另外,亲缘物种蛋白质序列同源性较高,在胰蛋白酶酶切过程中会出现错切或漏切等现象;以上因素都可能造成特征肽段在其他物种中出现干扰。

为确保所选肽段的特异性,本工作首先考察了骆驼奶特征肽段在牛奶、水牛奶、牦牛奶、山羊奶、绵羊奶、驴奶和马奶7个物种奶等质量混合液中的检出情况。检测结果如图5a所示,骆驼的4条特征肽段均未检出。本工作还考察了其他7个物种特征肽段在骆驼奶中的检出情况。结果如图5b所示,7个物种的特征肽段在骆驼奶中均未检出,其中检测到了个别肽段的单个离子对的色谱峰,但保留时间和对应肽段并不相同,为干扰离子峰,未出现保留时间和至少2个离子对相符合的情况。说明本工作中筛选出的特征肽段可以用于骆驼奶及其制品中的动物源性成分的测定。

**图5:**
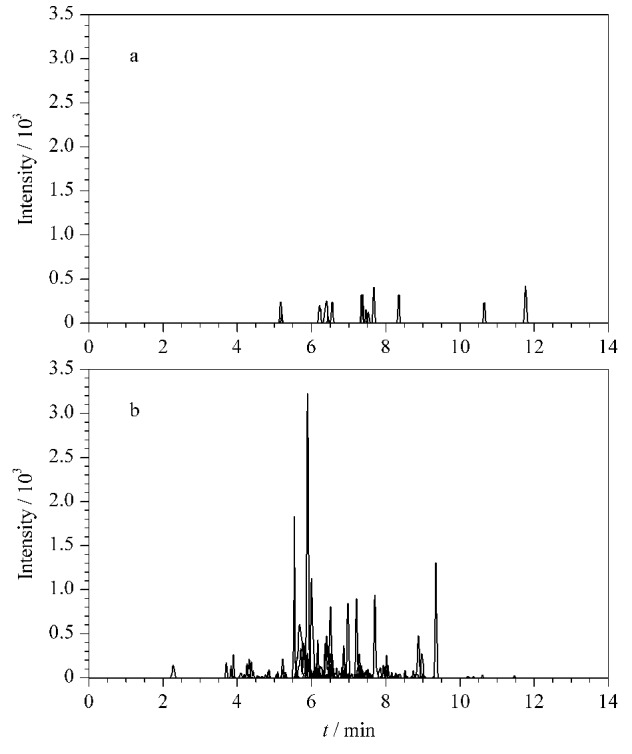
肽段特异性考察的提取离子流色谱图

### 2.5 掺假标准曲线的绘制

牛奶和山羊奶作为国内较普遍的两种奶源,其乳品成本相对较低,通常被掺入骆驼奶中以牟取经济利益,因此本工作对骆驼奶及奶粉进行了掺假定量研究。

向骆驼奶中掺入0、2.5%、5%、10%、25%、50%、75%、100%(质量分数)的牛奶或山羊奶,向骆驼奶粉中掺入上述相同质量分数的牛奶粉或山羊奶粉。以测量的每个混合样品中牛或山羊的特征肽段的峰面积为纵坐标,以添加的掺假物的质量分数为横坐标,绘制掺假定量标准曲线。如表2所示,掺假的标准曲线具有良好的线性,相关系数(*r*^2^)均大于0.99。以3倍和10倍信噪比(*S/N*)确定方法的检出限(LOD)和定量限(LOQ),最终确定骆驼奶中牛奶和山羊奶的掺假检出限分别为0.35%和0.49%,掺假定量限分别为1.20%和1.69%;骆驼奶粉中牛奶粉和山羊奶粉的掺假检出限分别为0.68%和0.73%,掺假定量限分别为1.65%和2.45%,见表2。

**表2:** 骆驼奶(粉)中掺入牛奶(粉)/山羊奶(粉)的定量肽段的线性方程、相关系数、检出限和定量限

Species	Peptide	Matrix	Linear equation	r^2^	LOD/%	LOQ/%
Bos taurus	SPAQILQWQVLSNTVPAK	milk	Y=35471.49X+1428.08	0.9998	0.35	1.20
		milk powder	Y=51022.83X+140196.35	0.9956	0.68	1.65
Capra hircus	FPQYLQYPYQGPIVLNPWDQVK	milk	Y=4490.73X+1362.76	0.9975	0.49	1.69
		milk powder	Y=5723.63X-9305.34	0.9965	0.73	2.45

Y: peak area; X: mass fraction, %.

在骆驼奶中分别添加质量分数为2.5%、5%和25%的牛奶和山羊奶,在骆驼奶粉中中分别添加质量分数为2.5%、5%和25%的牛奶粉和山羊奶粉,采用标准曲线定量计算加标回收率。定量肽段的平均回收率范围为85.8%~106.9%,日内精密度≤8.4%,日间精密度≤11.7%(*n*=3)。

### 2.6 掺假验证及实际样品检测

为考察掺假定量方法的准确性,本工作分别验证了将骆驼奶、牛奶、山羊奶,以及骆驼奶粉、牛奶粉、山羊奶粉均按照质量比1∶1∶1的比例分别混合后的定量检测结果。以家牛的特征肽段SPAQILQWQVLSNTVPAK(990.5^2+^→315.2^+^)和山羊的特征肽段FPQYLQYPYQGPIVLNPWDQVK(898.5^2+^→772.4^+^)为靶标进行检测。结果表明,液态奶混合样品中骆驼奶、牛奶和山羊奶的定量检测结果分别为38.7%、31.0%、30.3%;奶粉混合样品中骆驼奶粉、牛奶粉和山羊奶粉的定量检测结果分别为36.3%、32.2%、31.5%。液态奶和奶粉的掺杂检测结果均与理论值接近。因此,该方法可以为骆驼奶及奶粉掺假检测提供一定的技术支持。

本工作选择11个市售样品进行了实际样品的检测分析,其中包括5个驼乳及6个驼乳粉。测定结果表明,有10个样品仅检测出骆驼特征肽段,而1个驼奶粉样品中不仅检出了骆驼的特征肽段,还检出了家牛的特征肽段。经核对其配料表,发现这款奶粉含有全脂乳粉,但未标注其来源。因此,推断家牛的特征肽段来源于全脂乳粉。该实验也表明了建立该鉴别方法的必要性和现实意义。

**表3:** 11种市售样品的检测结果

Sample No.	Sample name	Detected species							
		Camelus	Bos taurus	Bubalus bubalis	Bos grunniens/ Bos mutus	Capra hircus	Ovis aries	Equus asinus	Equus caballus
1	camel milk 1	√	×	×	×	×	×	×	×
2	camel milk 2	√	×	×	×	×	×	×	×
3	camel milk 3	√	×	×	×	×	×	×	×
4	camel milk 4	√	×	×	×	×	×	×	×
5	camel milk 5	√	×	×	×	×	×	×	×
6	camel milk powder 1	√	×	×	×	×	×	×	×
7	camel milk powder 2	√	√	×	×	×	×	×	×
8	camel milk powder 3	√	×	×	×	×	×	×	×
9	camel milk powder 4	√	×	×	×	×	×	×	×
10	camel milk powder 5	√	×	×	×	×	×	×	×
11	camel milk powder 6	√	×	×	×	×	×	×	×

## 3 结论

本研究基于蛋白质组学技术,将UHPLC-Q/Exactive-HRMS和UHPLC-QqQ-MS/MS相结合,共筛选出8个物种的22条特征肽段。基于此,建立了以特征肽为分析对象的骆驼奶/奶粉的掺假鉴别和定量测定方法,并对11个实际样品进行检测,以确保标签的真实性。所开发的方法,具有高灵敏、高通量和可重复性等优点,有望为骆驼乳及其制品掺假检测提供一种有力的技术平台。
